# 2-Amino-6-methyl­pyridinium 2,2,2-tri­chloro­acetate

**DOI:** 10.1107/S1600536814004553

**Published:** 2014-03-05

**Authors:** K. Syed Suresh Babu, G. Peramaiyan, M. NizamMohideen, R. Mohan

**Affiliations:** a Department of Physics, Presidency College (Autonomous), Chennai 600 005, Tamil Nadu, India; b Department of Physics, The New College (Autonomous), Chennai 600 014, Tamil Nadu, India

## Abstract

In the asymmetric unit of the title mol­ecular salt, C_6_H_9_N_2_^+^·C_2_Cl_3_O_2_^−^, there are two independent 2-amino-6-methyl­pyridinium cations and two independent tri­chloro­acetate anions. The pyridine N atom of the 2-amino-6-methyl­pyridine mol­ecule is protonated and the geometries of these cations reveal amine–imine tautomerism. Both protonated 2-amino-6-methyl­pyridinium cations are essentially planar [maximum deviations = 0.026 (2) and 0.012 (2) Å]. In the crystal, the protonated N atom and the 2-amino group of the cation are hydrogen bonded to the carboxyl­ate O atoms of the anion *via* a pair of N—H⋯O hydrogen bonds, forming an *R*_2_^2^(8) ring motif. These motifs are connected *via* N—H⋯O and C—H⋯O hydrogen bonds to form slabs parallel to (101).

## Related literature   

For applications of pyridinium derivatives, see: Akkurt *et al.* (2005 ▶). For pyridine derivatives as templating agents, see: Desiraju (2001 ▶); Jeffrey (1997 ▶). For details of 2-amino­pyridine and its derivatives, see: Katritzky *et al.* (1996 ▶); Tomaru *et al.* (1991 ▶). For bond lengths and angles in similar structures, see: Jin, Shun *et al.* (2005 ▶); Feng *et al.* (2007 ▶); Nahringbauer & Kvick (1977 ▶). For other 2-amino­pyridinium structures, see: Jin *et al.* (2000 ▶, 2001 ▶); Jin, Tu *et al.* (2005 ▶). For studies on the tautomeric forms of 2-amino­pyridine systems, see: Ishikawa *et al.* (2002 ▶). For hydrogen-bond motifs, see: Bernstein *et al.* (1995 ▶).
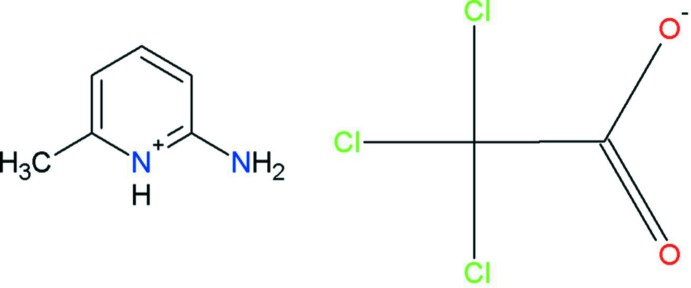


## Experimental   

### 

#### Crystal data  

C_6_H_9_N_2_^+^·C_2_Cl_3_O_2_^−^
*M* *_r_* = 271.52
Monoclinic, 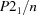

*a* = 11.6376 (5) Å
*b* = 14.6648 (6) Å
*c* = 13.9100 (6) Å
β = 96.024 (1)°
*V* = 2360.81 (17) Å^3^
*Z* = 8
Mo *K*α radiation
μ = 0.76 mm^−1^
*T* = 293 K
0.35 × 0.30 × 0.30 mm

#### Data collection  

Bruker Kappa APEXII CCD diffractometer
Absorption correction: multi-scan (*SADABS*; Sheldrick, 2004 ▶) *T*_min_ = 0.777, *T*_max_ = 0.805
39609 measured reflections
5784 independent reflections
3922 reflections with *I* > 2σ(*I*)
*R*_int_ = 0.032

#### Refinement  

*R*[*F* ^2^ > 2σ(*F* ^2^)] = 0.046
*wR*(*F* ^2^) = 0.129
*S* = 1.04
5784 reflections
297 parameters
6 restraints
H atoms treated by a mixture of independent and constrained refinement
Δρ_max_ = 0.66 e Å^−3^
Δρ_min_ = −0.64 e Å^−3^

### 

Data collection: *APEX2* (Bruker, 2004 ▶); cell refinement: *APEX2* and *SAINT* (Bruker, 2004 ▶); data reduction: *SAINT* and *XPREP* (Bruker, 2004 ▶); program(s) used to solve structure: *SHELXS97* (Sheldrick, 2008 ▶); program(s) used to refine structure: *SHELXL97* (Sheldrick, 2008 ▶); molecular graphics: *ORTEP-3 for Windows* (Farrugia, 2012 ▶) and *Mercury* (Macrae *et al.*, 2008 ▶); software used to prepare material for publication: *WinGX* (Farrugia, 2012 ▶) and *PLATON* (Spek, 2009 ▶).

## Supplementary Material

Crystal structure: contains datablock(s) global, I. DOI: 10.1107/S1600536814004553/su2705sup1.cif

Structure factors: contains datablock(s) I. DOI: 10.1107/S1600536814004553/su2705Isup2.hkl

Supporting information file. DOI: 10.1107/S1600536814004553/su2705Isup3.cml

CCDC reference: 988938

Additional supporting information:  crystallographic information; 3D view; checkCIF report

## Figures and Tables

**Table 1:** Hydrogen-bond geometry (Å, °)

*D*—H⋯*A*	*D*—H	H⋯*A*	*D*⋯*A*	*D*—H⋯*A*
N1—H1*A*⋯O3^i^	0.90 (1)	1.96 (1)	2.856 (3)	172 (3)
N2—H2*B*⋯O4^i^	0.89 (1)	1.95 (1)	2.825 (3)	167 (3)
N2—H2*A*⋯O1	0.89 (1)	2.11 (1)	2.982 (3)	167 (3)
N3—H3*A*⋯O2	0.90 (1)	1.84 (1)	2.729 (2)	175 (3)
N4—H4*B*⋯O1	0.88 (1)	2.06 (1)	2.929 (3)	167 (3)
N4—H4*A*⋯O3^ii^	0.88 (1)	2.29 (2)	3.096 (3)	152 (3)
C6—H6*C*⋯O1^iii^	0.96	2.50	3.413 (4)	158
C11—H11⋯O4^iv^	0.93	2.50	3.371 (4)	157

Symmetry codes: (i) 
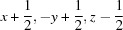
; (ii) 
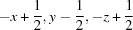
; (iii) 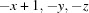; (iv) 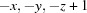.

**Fig. 1.**
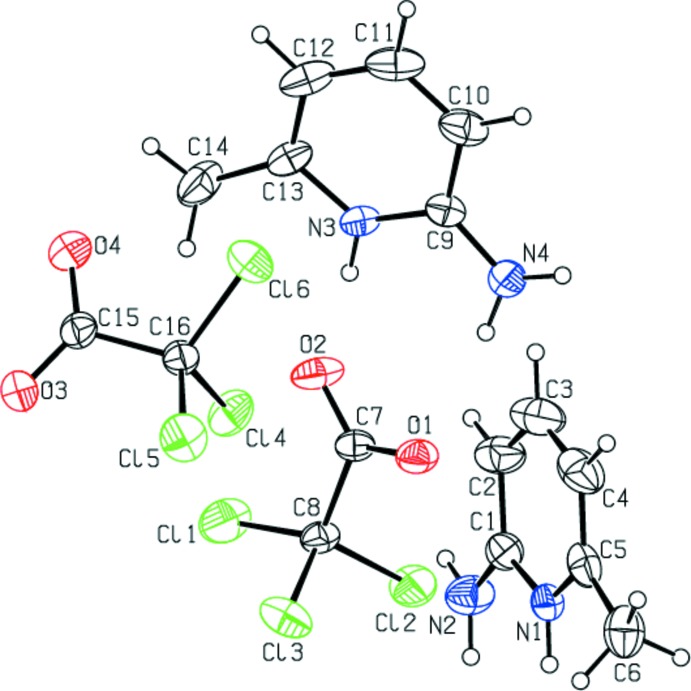
A view of the molecular structure of the two independent trichloroacetate anions and the two independent 2-amino-6-methylpyridinium cations of the title salt. Displacement ellipsoids are drawn at the 30% probability level.

**Fig. 2.**
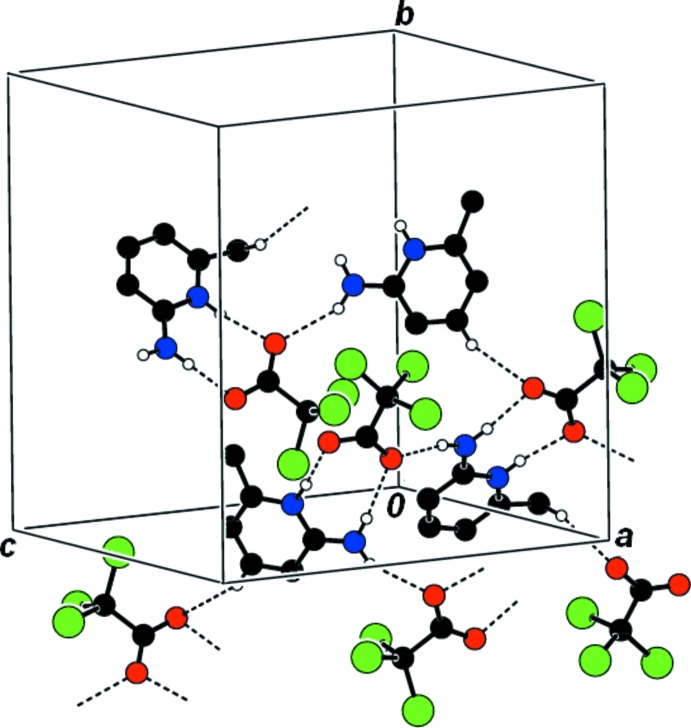
The crystal packing of the title compound, viewed normal to (101). The N—H···O and C—H···O hydrogen bonds are shown as dashed lines (see Table 1 for details; H atoms not involved in hydrogen bonding have been omitted for clarity).

## supplementary crystallographic information

### 1. Comment

2-Aminopyridine and its derivatives play an important role in heterocyclic chemistry (Katritzky *et al.*, 1996). Pyridine heterocycles and their derivatives are present in many large molecules having photo-chemical, electro-chemical and catalytic applications. Some pyridine derivatives possess nonlinear optical (NLO) properties (Tomaru *et al.*, 1991) and often possess antibacterial and antifungal activities (Akkurt *et al.*, 2005). The use of pyridine derivatives as templating agents for the self-assembly of organic-inorganic supramolecular materials has been widely studied (Desiraju, 2001). They are often involved in hydrogen bonding (Jeffrey, 1997). In order to further study the hydrogen bonding in these systems, the synthesis and structure of the title salt is presented herein.

The asymmetric unit of title compound, Fig. 1, consists of two crystallographically independent protonated 2-amino-6-methylpyridinium cation and trichloroacetate anion. The pyridinium rings are almost planar with mean deviations of only 0.011 (2) and 0.007 (2) Å for atoms N1 and N3, respectively. The dihedral angle between a 2-amino-6-methylpyridinium cation and trichloroacetate anion group is 18.3 (2) and 47.7 (2)° for the both molecules respectively. In both the molecules, the protonated 2-amino-6-methylpyridinium cations are essentially planar, with a maximum deviation of 0.026 (2) Å for atom N1 and 0.012 (2) Å for atom N3.

In the cations, the N2—C1 [1.324 (4) Å] and N4—C9 bonds [1.335 (3) Å] are shorter than the N1—C1 [1.357 (3) Å], N1—C5 [1.361 (3) Å], N3—C9[1.341 (3) Å] and N3—C13[1.360 (3) Å] bonds, and the C1—C2 [1.394 (4) Å], C3—C4 [1.401 (5) Å], C9—C10 [1.404 (3) Å] and C11—C12 [1.385 (5) Å] bonds are significantly longer than bonds C2—C3 [1.346 (5) Å], C4—C5 [1.360 (4) Å], C10—C11 [1.350 (5) Å] and C12—C13 [1.362 (4) Å]. This are similar to the bond distances observed previously for the amino-methylpridinium cation (Jin, Shun *et al.*, 2005; Feng *et al.*, 2007). In contrast, in the solid state structure of amino-methylpridinium, the exocyclic N—C bond is clearly longer than that in the ring (Nahringbauer & Kvick, 1977). The geometric features of amino-methylpridinium cation (N1/N2/C1/C6 and N3/N4/C9—C13) resemble those observed in other 2-aminopyridinium structures (Jin *et al.*, 2000; Jin *et al.*, 2001; Jin, Tu *et al.*, 2005) that are believed to be involved in amine-imine tautomerism (Ishikawa *et al.*, 2002). Similar features are also provided by the cation amino-methylpridinium (N3/N4/C7/C12). However,a previous study shows that a pyridinium cation always possesses an expanded angle of C—N—C [C1N1-C5 [123.7 (2)°] and C9—N3—C13 [124.6 (2)°] in comparison with the parent pyridine (Jin, Shun *et al.*, 2005).

In the crystal, the protonated atoms (N1 and N3) and a nitrogen atom of the 2-amino group (N2 and N4) are hydrogen bonded to the carboxylate oxygen atoms (O1, O2, O3 and O4) *via* pairs of intermolecular N—H—O [(N1—H1A···O3 and N3—H3A—O2) and (N2—H2B···O4 and N4—H4B···O1)] hydrogen bonds (Fig. 2 and Table 1), forming an *R*_2_^2^(8) ring motif (Bernstein *et al.*, 1995). Furthermore, these motifs are connected *via* N2—H2A···O1, N4—H4A···O3 and C—H···O hydrogen bonds to form slabs parallel with (101) [Fig. 2 and Table 1].

### 2. Experimental

Crystals of the title compound were obtained by slow evaporation of a 1:1 mol. mixture of 2-Amino-6-methylpyridine and trichloroacetic acid in methanol at room temperature.

### 3. Refinement

N-bound H atoms were located in a difference Fourier map and refined with distance restraints: N-H = 0.89 (1) and 0.90 (1) Å for NH_2_ and NH H atoms, respectively; N—H distances of the NH_2_ groups were restrained to be equal. The C-bound H atoms were positioned geometrically and refined using a riding model: C—H = 0.93–0.96 Å with *U*_iso_(H) = 1.5U_eq_(C-methyl) and = 1.2U_eq_(C) for other H atoms. A rotating group model was used for the methyl group.

### Crystal data

**Table:**

C_6_H_9_N_2_^+^·C_2_Cl_3_O_2_^−^	*F*(000) = 1104
*M**_r_* = 271.52	*D*_x_ = 1.528 Mg m^−^^3^
Monoclinic, *P*2_1_/*n*	Mo *K*α radiation, λ = 0.71073 Å
Hall symbol: -P 2yn	Cell parameters from 5784 reflections
*a* = 11.6376 (5) Å	θ = 2.0–28.1°
*b* = 14.6648 (6) Å	µ = 0.76 mm^−^^1^
*c* = 13.9100 (6) Å	*T* = 293 K
β = 96.024 (1)°	Block, colourless
*V* = 2360.81 (17) Å^3^	0.35 × 0.30 × 0.30 mm
*Z* = 8	

### Data collection

**Table:**

Bruker Kappa APEXII CCD diffractometer	5784 independent reflections
Radiation source: fine-focus sealed tube	3922 reflections with *I* > 2σ(*I*)
Graphite monochromator	*R*_int_ = 0.032
ω and φ scans	θ_max_ = 28.3°, θ_min_ = 2.0°
Absorption correction: multi-scan (*SADABS*; Sheldrick, 2004)	*h* = −15→12
*T*_min_ = 0.777, *T*_max_ = 0.805	*k* = −19→19
39609 measured reflections	*l* = −16→18

### Refinement

**Table:**

Refinement on *F*^2^	Primary atom site location: structure-invariant direct methods
Least-squares matrix: full	Secondary atom site location: difference Fourier map
*R*[*F*^2^ > 2σ(*F*^2^)] = 0.046	Hydrogen site location: inferred from neighbouring sites
*wR*(*F*^2^) = 0.129	H atoms treated by a mixture of independent and constrained refinement
*S* = 1.04	*w* = 1/[σ^2^(*F*_o_^2^) + (0.0512*P*)^2^ + 1.4785*P*] where *P* = (*F*_o_^2^ + 2*F*_c_^2^)/3
5784 reflections	(Δ/σ)_max_ < 0.001
297 parameters	Δρ_max_ = 0.66 e Å^−^^3^
6 restraints	Δρ_min_ = −0.64 e Å^−^^3^

### Special details

**Table:**

Geometry. All e.s.d.'s (except the e.s.d. in the dihedral angle between two l.s. planes) are estimated using the full covariance matrix. The cell e.s.d.'s are taken into account individually in the estimation of e.s.d.'s in distances, angles and torsion angles; correlations between e.s.d.'s in cell parameters are only used when they are defined by crystal symmetry. An approximate (isotropic) treatment of cell e.s.d.'s is used for estimating e.s.d.'s involving l.s. planes.
Refinement. Refinement of *F*^2^ against ALL reflections. The weighted *R*-factor *wR* and goodness of fit *S* are based on *F*^2^, conventional *R*-factors *R* are based on *F*, with *F* set to zero for negative *F*^2^. The threshold expression of *F*^2^ > σ(*F*^2^) is used only for calculating *R*-factors(gt) *etc*. and is not relevant to the choice of reflections for refinement. *R*-factors based on *F*^2^ are statistically about twice as large as those based on *F*, and *R*- factors based on ALL data will be even larger.

### Fractional atomic coordinates and isotropic or equivalent isotropic displacement parameters (Å^2^)

**Table:**

	*x*	*y*	*z*	*U*_iso_*/*U*_eq_	
C1	0.3813 (2)	0.07767 (18)	0.03971 (18)	0.0507 (6)	
C2	0.3271 (3)	0.0212 (2)	0.1018 (2)	0.0713 (8)	
H2	0.3343	0.0326	0.1679	0.086*	
C3	0.2640 (3)	−0.0500 (3)	0.0649 (3)	0.0852 (10)	
H3	0.2284	−0.0880	0.1063	0.102*	
C4	0.2509 (3)	−0.0681 (2)	−0.0346 (3)	0.0751 (9)	
H4	0.2071	−0.1176	−0.0588	0.090*	
C5	0.3029 (2)	−0.01251 (17)	−0.0952 (2)	0.0549 (6)	
C6	0.2932 (3)	−0.0214 (2)	−0.2021 (2)	0.0737 (9)	
H6A	0.2729	0.0366	−0.2311	0.111*	
H6B	0.2345	−0.0653	−0.2226	0.111*	
H6C	0.3658	−0.0412	−0.2216	0.111*	
C7	0.49848 (19)	0.20079 (16)	0.35230 (16)	0.0429 (5)	
C8	0.5578 (2)	0.29325 (16)	0.33022 (17)	0.0464 (5)	
C9	0.3521 (2)	−0.03512 (16)	0.41212 (17)	0.0476 (5)	
C10	0.2838 (3)	−0.1102 (2)	0.4323 (2)	0.0663 (8)	
H10	0.2952	−0.1667	0.4044	0.080*	
C11	0.2013 (3)	−0.0996 (2)	0.4928 (2)	0.0753 (9)	
H11	0.1557	−0.1492	0.5060	0.090*	
C12	0.1831 (3)	−0.0162 (2)	0.5356 (2)	0.0710 (9)	
H12	0.1254	−0.0100	0.5766	0.085*	
C13	0.2502 (2)	0.0565 (2)	0.51737 (16)	0.0544 (6)	
C14	0.2420 (3)	0.1494 (2)	0.5583 (2)	0.0767 (9)	
H14A	0.3029	0.1580	0.6096	0.115*	
H14B	0.1686	0.1565	0.5832	0.115*	
H14C	0.2490	0.1939	0.5086	0.115*	
C15	0.0091 (2)	0.26412 (17)	0.33994 (19)	0.0491 (6)	
C16	0.0641 (2)	0.19779 (16)	0.26897 (18)	0.0460 (5)	
N1	0.36821 (17)	0.05787 (13)	−0.05609 (15)	0.0459 (4)	
N3	0.33292 (17)	0.04379 (13)	0.45650 (13)	0.0437 (4)	
N2	0.4457 (2)	0.14867 (19)	0.06970 (17)	0.0668 (6)	
N4	0.4353 (2)	−0.03672 (15)	0.35294 (18)	0.0605 (6)	
O1	0.48960 (16)	0.14569 (12)	0.28478 (12)	0.0549 (4)	
O2	0.46713 (18)	0.19317 (14)	0.43335 (12)	0.0660 (5)	
O3	−0.01711 (18)	0.34018 (12)	0.30739 (14)	0.0637 (5)	
O4	0.0005 (2)	0.23281 (15)	0.42110 (15)	0.0791 (6)	
Cl1	0.55673 (9)	0.37373 (6)	0.42317 (6)	0.0896 (3)	
Cl2	0.70242 (6)	0.27091 (5)	0.31008 (6)	0.0710 (2)	
Cl3	0.48451 (7)	0.34145 (5)	0.22382 (6)	0.0752 (2)	
Cl4	0.21459 (6)	0.22009 (6)	0.28120 (6)	0.0697 (2)	
Cl5	0.01042 (7)	0.21709 (6)	0.14738 (5)	0.0719 (2)	
Cl6	0.04405 (9)	0.08231 (5)	0.29620 (7)	0.0861 (3)	
H4B	0.463 (2)	0.0160 (11)	0.3358 (19)	0.059 (8)*	
H2B	0.474 (2)	0.1848 (16)	0.0269 (17)	0.064 (9)*	
H4A	0.447 (3)	−0.0864 (14)	0.320 (2)	0.082 (10)*	
H2A	0.455 (3)	0.157 (2)	0.1332 (8)	0.082 (10)*	
H1A	0.403 (2)	0.0949 (15)	−0.0955 (16)	0.057 (8)*	
H3A	0.379 (2)	0.0908 (13)	0.446 (2)	0.061 (8)*	

### Atomic displacement parameters (Å^2^)

**Table:**

	*U*^11^	*U*^22^	*U*^33^	*U*^12^	*U*^13^	*U*^23^
C1	0.0481 (14)	0.0549 (14)	0.0483 (13)	0.0081 (11)	0.0017 (10)	0.0075 (11)
C2	0.0690 (19)	0.084 (2)	0.0605 (17)	−0.0020 (16)	0.0039 (14)	0.0247 (16)
C3	0.078 (2)	0.083 (2)	0.095 (3)	−0.0068 (19)	0.0106 (19)	0.039 (2)
C4	0.0634 (19)	0.0526 (16)	0.108 (3)	−0.0103 (14)	0.0013 (17)	0.0084 (17)
C5	0.0451 (14)	0.0417 (12)	0.0764 (18)	0.0072 (10)	−0.0007 (12)	−0.0040 (12)
C6	0.076 (2)	0.0650 (18)	0.079 (2)	0.0008 (15)	0.0022 (16)	−0.0237 (16)
C7	0.0384 (11)	0.0478 (12)	0.0423 (12)	−0.0102 (10)	0.0031 (9)	0.0052 (10)
C8	0.0464 (13)	0.0488 (13)	0.0453 (12)	−0.0116 (10)	0.0105 (10)	0.0016 (10)
C9	0.0539 (14)	0.0453 (13)	0.0428 (12)	−0.0052 (11)	0.0019 (10)	0.0071 (10)
C10	0.079 (2)	0.0514 (15)	0.0682 (18)	−0.0218 (14)	0.0046 (15)	0.0071 (13)
C11	0.077 (2)	0.084 (2)	0.0644 (18)	−0.0401 (18)	0.0034 (15)	0.0191 (16)
C12	0.0600 (17)	0.104 (2)	0.0517 (15)	−0.0272 (17)	0.0167 (13)	0.0075 (16)
C13	0.0502 (14)	0.0788 (18)	0.0352 (12)	−0.0098 (13)	0.0087 (10)	0.0016 (12)
C14	0.082 (2)	0.092 (2)	0.0611 (17)	−0.0084 (18)	0.0311 (16)	−0.0186 (17)
C15	0.0401 (12)	0.0493 (14)	0.0595 (15)	−0.0035 (10)	0.0123 (10)	−0.0009 (11)
C16	0.0422 (12)	0.0440 (12)	0.0525 (13)	−0.0031 (10)	0.0074 (10)	0.0012 (10)
N1	0.0439 (11)	0.0427 (10)	0.0513 (11)	0.0070 (9)	0.0057 (9)	0.0027 (9)
N3	0.0475 (11)	0.0494 (11)	0.0349 (9)	−0.0116 (9)	0.0073 (8)	0.0031 (8)
N2	0.0811 (17)	0.0736 (16)	0.0452 (13)	−0.0144 (13)	0.0042 (12)	−0.0005 (12)
N4	0.0733 (16)	0.0448 (12)	0.0675 (14)	−0.0029 (11)	0.0263 (12)	−0.0012 (11)
O1	0.0684 (11)	0.0488 (9)	0.0492 (10)	−0.0155 (8)	0.0144 (8)	−0.0021 (8)
O2	0.0833 (14)	0.0737 (12)	0.0437 (10)	−0.0383 (11)	0.0188 (9)	−0.0022 (9)
O3	0.0745 (13)	0.0496 (10)	0.0696 (12)	0.0085 (9)	0.0205 (10)	0.0019 (9)
O4	0.1060 (18)	0.0731 (13)	0.0642 (13)	0.0115 (12)	0.0377 (12)	0.0076 (10)
Cl1	0.1227 (8)	0.0727 (5)	0.0798 (5)	−0.0399 (5)	0.0406 (5)	−0.0284 (4)
Cl2	0.0438 (4)	0.0737 (5)	0.0975 (6)	−0.0119 (3)	0.0168 (3)	0.0099 (4)
Cl3	0.0823 (5)	0.0593 (4)	0.0805 (5)	−0.0066 (4)	−0.0083 (4)	0.0246 (4)
Cl4	0.0392 (3)	0.0947 (6)	0.0764 (5)	0.0005 (3)	0.0112 (3)	−0.0015 (4)
Cl5	0.0810 (5)	0.0774 (5)	0.0538 (4)	0.0001 (4)	−0.0092 (3)	−0.0080 (3)
Cl6	0.1113 (7)	0.0446 (4)	0.1068 (7)	−0.0056 (4)	0.0323 (5)	0.0062 (4)

### Geometric parameters (Å, º)

**Table:**

C1—N2	1.324 (4)	C10—C11	1.350 (5)
C1—N1	1.357 (3)	C10—H10	0.9300
C1—C2	1.394 (4)	C11—C12	1.385 (5)
C2—C3	1.346 (5)	C11—H11	0.9300
C2—H2	0.9300	C12—C13	1.362 (4)
C3—C4	1.401 (5)	C12—H12	0.9300
C3—H3	0.9300	C13—N3	1.360 (3)
C4—C5	1.360 (4)	C13—C14	1.483 (4)
C4—H4	0.9300	C14—H14A	0.9600
C5—N1	1.361 (3)	C14—H14B	0.9600
C5—C6	1.485 (4)	C14—H14C	0.9600
C6—H6A	0.9600	C15—O3	1.230 (3)
C6—H6B	0.9600	C15—O4	1.232 (3)
C6—H6C	0.9600	C15—C16	1.569 (3)
C7—O2	1.226 (3)	C16—Cl6	1.756 (2)
C7—O1	1.235 (3)	C16—Cl5	1.763 (3)
C7—C8	1.567 (3)	C16—Cl4	1.772 (2)
C8—Cl1	1.752 (2)	N1—H1A	0.898 (10)
C8—Cl2	1.765 (3)	N3—H3A	0.896 (10)
C8—Cl3	1.776 (3)	N2—H2B	0.885 (10)
C9—N4	1.335 (3)	N2—H2A	0.886 (10)
C9—N3	1.341 (3)	N4—H4B	0.881 (10)
C9—C10	1.404 (3)	N4—H4A	0.879 (10)
			
N2—C1—N1	118.6 (2)	C10—C11—C12	121.4 (3)
N2—C1—C2	123.3 (3)	C10—C11—H11	119.3
N1—C1—C2	118.0 (3)	C12—C11—H11	119.3
C3—C2—C1	119.2 (3)	C13—C12—C11	119.7 (3)
C3—C2—H2	120.4	C13—C12—H12	120.2
C1—C2—H2	120.4	C11—C12—H12	120.2
C2—C3—C4	121.5 (3)	N3—C13—C12	117.7 (3)
C2—C3—H3	119.3	N3—C13—C14	116.3 (2)
C4—C3—H3	119.3	C12—C13—C14	126.0 (3)
C5—C4—C3	119.3 (3)	C13—C14—H14A	109.5
C5—C4—H4	120.3	C13—C14—H14B	109.5
C3—C4—H4	120.3	H14A—C14—H14B	109.5
C4—C5—N1	118.2 (3)	C13—C14—H14C	109.5
C4—C5—C6	125.2 (3)	H14A—C14—H14C	109.5
N1—C5—C6	116.6 (2)	H14B—C14—H14C	109.5
C5—C6—H6A	109.5	O3—C15—O4	129.4 (2)
C5—C6—H6B	109.5	O3—C15—C16	115.6 (2)
H6A—C6—H6B	109.5	O4—C15—C16	115.0 (2)
C5—C6—H6C	109.5	C15—C16—Cl6	112.94 (17)
H6A—C6—H6C	109.5	C15—C16—Cl5	112.14 (17)
H6B—C6—H6C	109.5	Cl6—C16—Cl5	108.66 (13)
O2—C7—O1	129.1 (2)	C15—C16—Cl4	106.82 (16)
O2—C7—C8	116.1 (2)	Cl6—C16—Cl4	108.04 (13)
O1—C7—C8	114.87 (19)	Cl5—C16—Cl4	108.03 (13)
C7—C8—Cl1	113.64 (16)	C1—N1—C5	123.7 (2)
C7—C8—Cl2	108.53 (17)	C1—N1—H1A	117.3 (18)
Cl1—C8—Cl2	108.85 (13)	C5—N1—H1A	118.9 (18)
C7—C8—Cl3	108.95 (16)	C9—N3—C13	124.6 (2)
Cl1—C8—Cl3	107.87 (14)	C9—N3—H3A	117.3 (18)
Cl2—C8—Cl3	108.92 (12)	C13—N3—H3A	118.1 (18)
N4—C9—N3	117.7 (2)	C1—N2—H2B	120 (2)
N4—C9—C10	124.9 (3)	C1—N2—H2A	115 (2)
N3—C9—C10	117.4 (2)	H2B—N2—H2A	125 (3)
C11—C10—C9	119.2 (3)	C9—N4—H4B	117.6 (19)
C11—C10—H10	120.4	C9—N4—H4A	120 (2)
C9—C10—H10	120.4	H4B—N4—H4A	120 (3)
			
N2—C1—C2—C3	−179.4 (3)	C11—C12—C13—N3	0.3 (4)
N1—C1—C2—C3	−0.2 (4)	C11—C12—C13—C14	−179.4 (3)
C1—C2—C3—C4	−0.6 (5)	O3—C15—C16—Cl6	−154.9 (2)
C2—C3—C4—C5	0.0 (5)	O4—C15—C16—Cl6	26.5 (3)
C3—C4—C5—N1	1.4 (4)	O3—C15—C16—Cl5	−31.7 (3)
C3—C4—C5—C6	−177.7 (3)	O4—C15—C16—Cl5	149.7 (2)
O2—C7—C8—Cl1	−5.5 (3)	O3—C15—C16—Cl4	86.5 (2)
O1—C7—C8—Cl1	174.28 (18)	O4—C15—C16—Cl4	−92.2 (2)
O2—C7—C8—Cl2	115.8 (2)	N2—C1—N1—C5	−179.0 (2)
O1—C7—C8—Cl2	−64.5 (2)	C2—C1—N1—C5	1.7 (4)
O2—C7—C8—Cl3	−125.8 (2)	C4—C5—N1—C1	−2.3 (4)
O1—C7—C8—Cl3	54.0 (3)	C6—C5—N1—C1	176.9 (2)
N4—C9—C10—C11	−179.3 (3)	N4—C9—N3—C13	178.8 (2)
N3—C9—C10—C11	1.5 (4)	C10—C9—N3—C13	−1.9 (4)
C9—C10—C11—C12	−0.3 (5)	C12—C13—N3—C9	1.0 (4)
C10—C11—C12—C13	−0.6 (5)	C14—C13—N3—C9	−179.3 (3)

### Hydrogen-bond geometry (Å, º)

**Table:**

*D*—H···*A*	*D*—H	H···*A*	*D*···*A*	*D*—H···*A*
N1—H1*A*···O3^i^	0.90 (1)	1.96 (1)	2.856 (3)	172 (3)
N2—H2*B*···O4^i^	0.89 (1)	1.95 (1)	2.825 (3)	167 (3)
N2—H2*A*···O1	0.89 (1)	2.11 (1)	2.982 (3)	167 (3)
N3—H3*A*···O2	0.90 (1)	1.84 (1)	2.729 (2)	175 (3)
N4—H4*B*···O1	0.88 (1)	2.06 (1)	2.929 (3)	167 (3)
N4—H4*A*···O3^ii^	0.88 (1)	2.29 (2)	3.096 (3)	152 (3)
C6—H6*C*···O1^iii^	0.96	2.50	3.413 (4)	158
C11—H11···O4^iv^	0.93	2.50	3.371 (4)	157

Symmetry codes: (i) *x*+1/2, −*y*+1/2, *z*−1/2; (ii) −*x*+1/2, *y*−1/2, −*z*+1/2; (iii) −*x*+1, −*y*, −*z*; (iv) −*x*, −*y*, −*z*+1.
